# Prognostic Factors of Patients With Transmural Advanced Gastric Carcinoma

**DOI:** 10.4021/gr2009.11.1322

**Published:** 2009-11-20

**Authors:** Hayrullah Derici, Ismail Yaman, Tugrul Tansug, Okay Nazli, Ali Dogan Bozdag, Ali Serdar Isguder

**Affiliations:** a Third Surgical Clinic of Atatürk Training and Research Hospital, Izmir, Turkey

**Keywords:** Gastric Cancer, Transmural, Advanced, Morbidity, Mortality, Survival

## Abstract

**Background:**

The purpose of this study is to evaluate perioperative morbidity, mortality and the prognostic factors that influence survival of the patients with transmural advanced gastric carcinoma after curative surgical therapy.

**Methods:**

Fifty patients with transmural advanced gastric adenocarcinoma underwent curative resection in our clinic. The records of the patients were reviewed and the prognostic factors such as age, gender, location and size of the tumor, type of surgery, blood transfusion, depth of tumor invasion, lymph node metastases, stage of the disease, grading, vascular invasion, lymph vessel invasion, characteristics of the tumor according to Lauren’s classification, and lymph node ratio were evaluated by using statistical methods.

**Results:**

In a total of 12 patients (24%) major morbidities developed, and five patients (10%) died. The overall survival rate was 48% at 1 year, 31% at 3 years, and 19% at 5 years. Lymph node metastases (P = 0.03), lymph vessel invasion (P = 0.001), blood transfusion (P = 0.021), and lymph node ratio (P = 0.006) were the prognostic features identified by univariate analysis. Among the multiple significant prognostic factors in the univariate analysis only one factor, lymph node ratio, proved to be independently significant in the multivariate analysis (RR: 4.47).

**Conclusions:**

Our data showed that we can expect a good survival for patients with a lymph node ratio less than 0.2.

## Introduction

Despite developments in diagnostic and therapeutic modalities, gastric cancer is still the second most common cause of cancer-related death throughout the world [1, 2]. The highest incidences of gastric cancer are reported from Eastern Asia, Eastern Europe, and South America [3]. The prognosis for gastric cancer patients remains poor, especially in advanced stages of the disease. There were about 158,000 deaths from stomach cancer in Europe in 2000 [4]. According to 1999 figures of State’s Statistics Institute, death from gastric cancer was 3.22/100.000 in Turkey [5]. A very rigorous screening program that detects patients in an early stage of the disease was developed in Japan. Up to 70% of all gastric cancers are diagnosed as early cancers in the East, but the rate of gastric cancers identified as early cancers accounts for only about 15% in the West [6].

Patients with transmural advanced gastric cancer (T2N0-T3N2) make up the largest group with uncertain outcome. Although the therapeutic results in this group are variable, the identifying prognostic factors are important to establish a therapeutic strategy [7]. This retrospective study was designed with the aim to evaluate perioperative morbidity, mortality and the prognostic factors that influence survival of the patients with transmural advanced gastric carcinoma after curative surgical therapy.

## Materials and Methods

One hundred and forty-five patients with primary gastric cancer underwent gastric resection in our clinic, between January 1997 and March 2004. Fifty patients (34.5%) with transmural advanced gastric cancer were selected for this retrospective study. Hospital records of the patients were reviewed for clinicopathological findings, morbidity, mortality, and survival. The patients with synchronous distant metastases, peritoneal carcinomatosis, gastric stump carcinoma, recurrent gastric cancer, and those that received noncurative resections (R_1_, R_2_) were excluded.

Preoperative work-up included upper gastrointestinal endoscopy with biopsy, abdominal ultrasound scan, and computed tomography. Diagnostic laparoscopy was selectively performed. Gastrectomy with an intent of D2 lymphadenectomy was performed as described by Nakajima and Kajitani [8]. Total gastrectomy was performed for tumors located on the proximal or middle portions of the stomach and subtotal gastrectomy for distal tumors with curative aim. Staging of the gastric cancer was made with reference to the TNM classification of malignant tumors by the International Union Against Cancer (UICC). The pN category which was established to evaluate different groups of lymph node metastases was used (pN0:0, pN1: 1-6, pN2: 7-15 and pN3: >15) [9]. The extent of lymph node dissection and lymph node ratio (the proportion of the number of positive nodes to the number of all resected nodes) are noted. The patients were put into two groups considering lymph node ratio as less than 0.2, and greater than or equal to 0.2.

The patients were followed up at regular intervals by outpatient visits and telephone interviews. The cases that died in the early postoperative period were excluded from the survival analysis. Survival curves were estimated using the Kaplan-Meier method and differences in survival were compared by the log-rank test. Survival was calculated from the date of operation to the date of death, or to the date of last follow-up for the surviving patients. Median survival time was given with their standard error (SE). Simultaneous associations of multiple variables were performed using the Cox proportional hazards regression model to estimate the simultaneous effect on overall survival. Association of the independent prognostic factors, which were age, gender, location and size of the tumor, type of surgery, blood transfusion, depth of tumor invasion (pT), lymph node metastases (pN), stage of the disease, grading, vascular invasion, lymph vessel invasion, characteristics of the tumor according to Lauren’s classification, and lymph node ratio with patients’ survival were evaluated by using univariate analysis methods. Independent variables that showed statistical significance in the univariate analysis were then entered in the multivariate analysis. The prognostic factors were compared by hazard ratio and the 95% confidence interval (CI). Stepwise selection was used to find the most significant factors. A P value of less than 0.05 was considered statistically significant for all patients. Statistical Package for Social Sciences (SPSS 10.0, Chicago, IL, USA) for Windows (Microsoft) was used for the statistical analyses.

## Results

Of the 50 patients, 30 (60%) were male and 20 (40%) were female, with a median age of 61 years (range 31 - 77 years). Total gastrectomy was performed in 25 patients and subtotal gastrectomy in the other 25. Histopathological examination of the resected tumors revealed that all of the cases were adenocarcinoma: 38% of them were intestinal type and 62% were diffuse type. The most common site of the primary lesion was antrum (25 cases, 50%). The majority of the tumors were pT3 (39 cases, 78%). All patients had received D2 lymphadenectomy. The median count of dissected lymph nodes was 34 ranging between 25 and 46. Distribution of the cases according to lymph node metastases was as follows: pN0:10 cases, pN1:28 cases, and pN2:12 cases.

Deaths during the first postoperative month were considered surgical mortality. A total of 16 major morbidities developed in 12 patients (24%). Anastomotic leakage was observed in five cases, duodenal stump dehiscence in three cases, abdominal abscess, pneumonia, and ileus in two cases for each, hemorrhage, and myocardial infarction in one case for each. Five patients (10%) died due to these complications and they were excluded from the survival analysis.

Thirty-six patients received postoperative concomitant radio-chemotherapy as adjuvant treatment. All of them completed the whole course of adjuvant treatment. Nine patients did not receive adjuvant therapy due to postoperative complications (four patients), or patient’s refusal to receive radio-chemotherapy (five patients).

Mean hospital stay was 13.5 days (8 - 40 days). Median follow-up was 25 months (range 2 - 74 months). During follow-up period 29 patients died of gastric cancer and 16 patients are still alive. The overall survival rate was 48% at 1 year, 31% at 3 years, and 19% at 5 years, and median survival time was 23.00 ± 2.51 months, 95% Cl (18.07 - 27.93). There was no significant difference in survival between the patients with pT2 and pT3 tumors, nor between stage III B disease and the other stages. Median survival time was 24.0 ± 2.45 months, 95% Cl (19.19 - 28.81), for the patients with a lymph node ratio less than 0.2; and 5.0 ± 2.0 months, 95% Cl (1.08 - 8.92), for those with a lymph node ratio greater or equal to 0.2 (P = 0.0012, log-rank test).

Univariate analysis was performed to evaluate the relationship between clinicopathological features and patients’ survival. Of the 14 clinical and pathological variables entered in the analysis, four were found to have significant influence. Lymph node metastases (P = 0.030), lymph vessel invasion (P = 0.001), blood transfusion (P = 0.021), and lymph node ratio (P = 0.006) were the prognostic features identified by univariate analysis. Evaluation of the prognostic factors for the cases and the results of univariate analysis are shown in Table 1. Among multiple significant prognostic factors in the univariate analysis, only one factor, lymph node ratio, proved to be independently significant in the multivariate analysis, risk ratio 4.47, 95% CI (1.168 - 17.117) (Table 2).

**Table 1:** Univariate Analysis of The Prognostic Factors In Patients With Transmural Gastric Cancer

Parameters	No. of patients		95% Confidence		Survival rate (%)			*P*†
		Median survival (month) ± SE	Interval	P*	1-year	3-years	5-years	
Age (years)				NS				NS
≤65	30	25.0 ± 7.25	27.88 – 48.77		52	40	20	
>65	15	16.0 ± 9.02	0 – 33.67		39	13	13	
Gender				NS				NS
Male	27	21.0 ± 3.44	14.25 – 27.75		44	25	25	
Female	18	27.0 ± 7.80	11.70 – 42.30		53	41	0	
Tumor location				NS				NS
Cardia	9	30.0 ± 7.28	15.73 – 44.27		49	32	0	
Corpus	11	18.0 ± 3.48	11.17 – 24.83		35	35	35	
Antrum	22	24.0 ± 3.03	18.07 – 29.93		55	35	35	
Diffuse	3	0	0		-	-	-	
Tumor size (cm)				NS				NS
>10 in diameter	24	24.0 ± 5.30	13.61 – 34.39		54	36	18	
≤10 in diameter	21	21.0 ± 2.28	16.53 – 25.47		43	28	28	
Depth of tumor invasion				NS				NS
pT2	11	62.0 ± 19.12	24.52 – 99.48		53	53	26	
pT3	34	18.0 ± 5.38	7.45 – 28.55		46	26	18	
Lymph node metastases				0.04				0.03
pN0	10	-	-		78	65	65	
pN1	25	21.0 ± 2.38	16.33 – 25.67		37	27	13	
pN2	10	16.0 ± 6.39	3.47 – 28.53		46	11	11	
Stage				NS				NS
I B	3	-	-		100	100	100	
II	16	21.0 ± 5.04	11.13 – 30.87		46	37	0	
III A	17	18.0 ± 4.84	8.52 – 27.48		38	25	25	
III B	9	24.0 ± 6.01	12.21 – 35.79		51	13	-	
Grading				0.017				NS
G1	6	-	-		100	100	100	
G2	6	21.0 ± 9.19	3.0 – 39.0		50	33	0	
G3	33	18.0 ± 3.39	11.36 – 24.64		38	19	19	
Type of Surgery				NS				NS
Subtotal gastrectomy	22	24.0 ± 3.03	18.07 – 29.93		55	35	35	
Total gastrectomy	23	20.0 ± 3.16	13.81 – 26.19		41	28	0	
Vascular invasion				NS				NS
Absent	26	25.0 ± 5.07	15.07 – 34.93		54	35	35	
Present	19	21.0 ± 2.05	16.99 – 25.01		39	26	9	
Lymph vessel invasion				0.0003				0.001
Absent	17	62.0 ± 22.96	17.0 – 107.0		80	64	22	
Present	28	15.0 ± 2.55	10.01 – 19.99		29	12	12	
Blood transfusions				0.0166				0.021
< 500 ml	27	27.0 ± 6.85	13.58 – 40.42		57	44	44	
≥ 500 ml	18	16.0 ± 5.63	4.97 – 27.03		32	13	4	
Lauren’s classification				NS				NS
Intestinal	16	25.0 ± 6.0	13.24 – 36.76		63	42	42	
Diffuse	29	18.0 ± 3.62	10.90 – 25.10		39	25	8	
Lymph node ratio				0.0012				0.006
LNR < 0.2	12	24.0 ± 2.45	19.19 – 28.81		52	34	20	
LNR ≥ 0.2	33	5.0 ± 2.0	1.08 – 8.92		-	-	-	
All patients	45	23.0 ± 2.51	18.07 – 27.93		48	31	19	

SE: Standard error, P *: Log-rank test, P†: Chi-square test, NS: Not significant

**Table 2:** Multivariate Analysis of the Independent Prognostic Factors

Parameters	RR	95% CI	*P*
Lymph node metastases			
(pN1 vs pN0)	1.11	0.285 – 4.341	0.877
(pN2 vs pN0)	1.46	0.282 – 7.544	0.652
Lymph vessel invasion (Extensive vs not extensive)	1.39	0.444 – 4.379	0.559
Blood transfusions (≥ 500 ml vs < 500 ml)	1.01	0.433 – 2.362	0.979
Lymph node ratio (≥ 0.2 vs < 0.2)	4.47	1.168 – 17.117	0.029

P: Cox’s proportional hazards model. CI: Confidence Interval. RR: Relative Risk.

**Figure 1:**
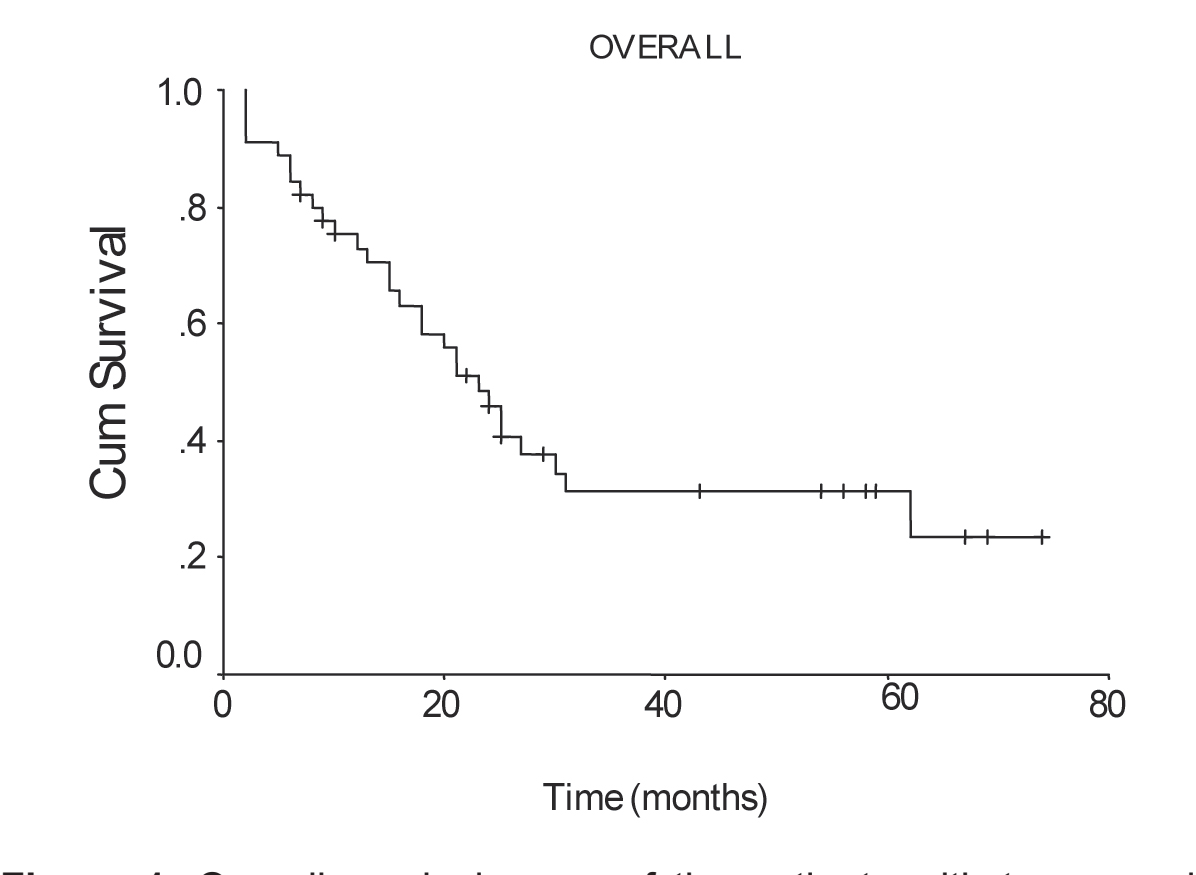
Overall survival curve of the patients with transmural gastric carcinoma calculated by the method of Kaplan-Meier.

**Figure 2:**
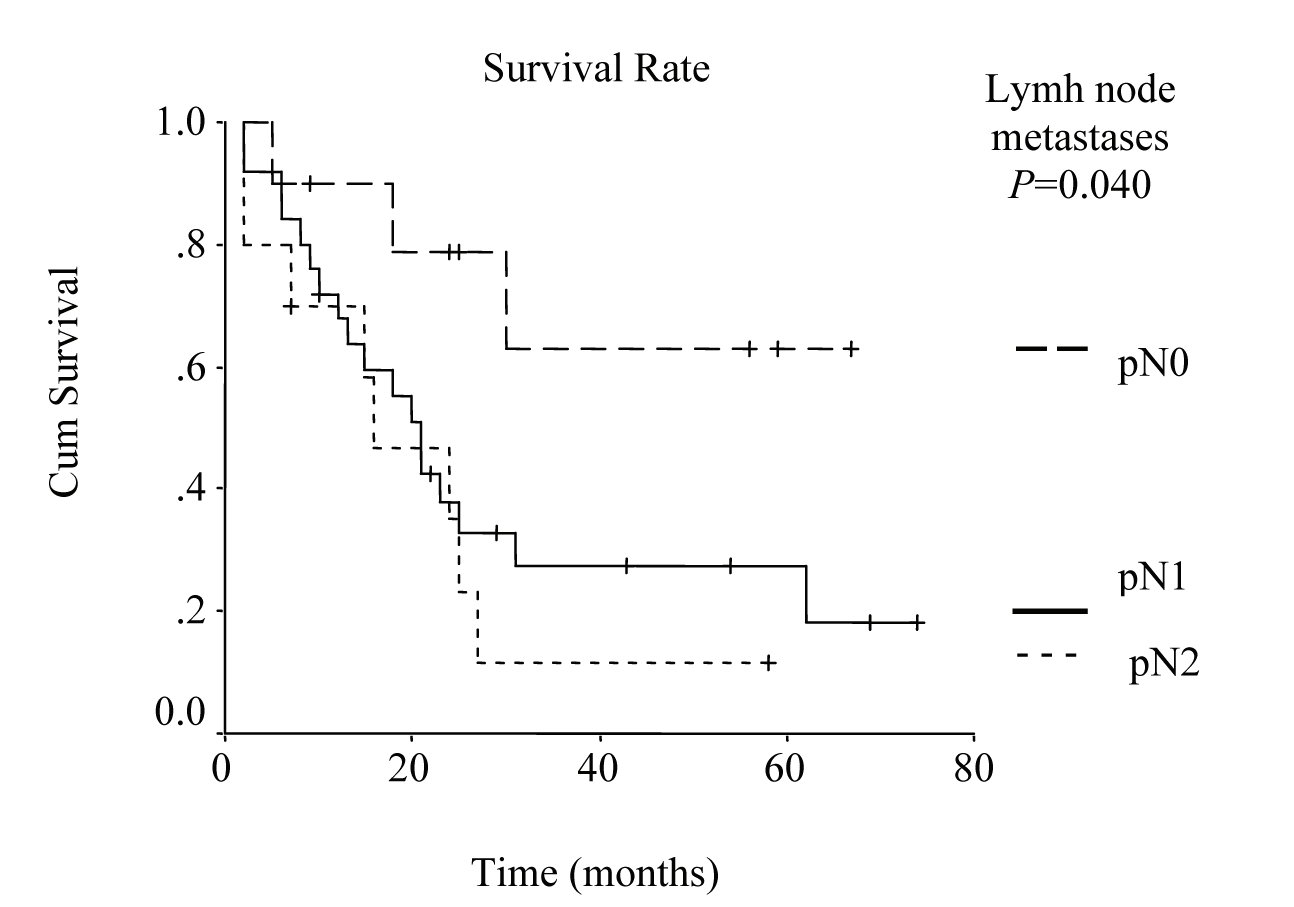
Survival according to nodal involvement. The survival rate for the patients with pN2 lymph node metastases was lower than the rate for pN1 and pN0 cases. (P = 0.0400, log-rank test).

**Figure 3:**
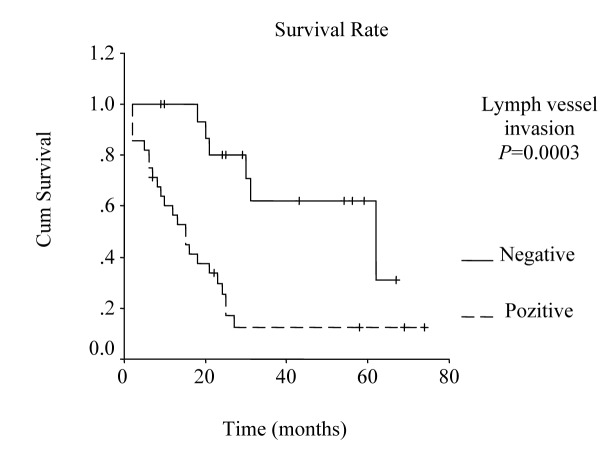
Survival according to lymph vessel invasion. The survival rate for patients with lymph vessel invasion was lower than the rate for the patients without lymph vessel invasion. (P = 0.0003, log-rank test).

**Figure 4:**
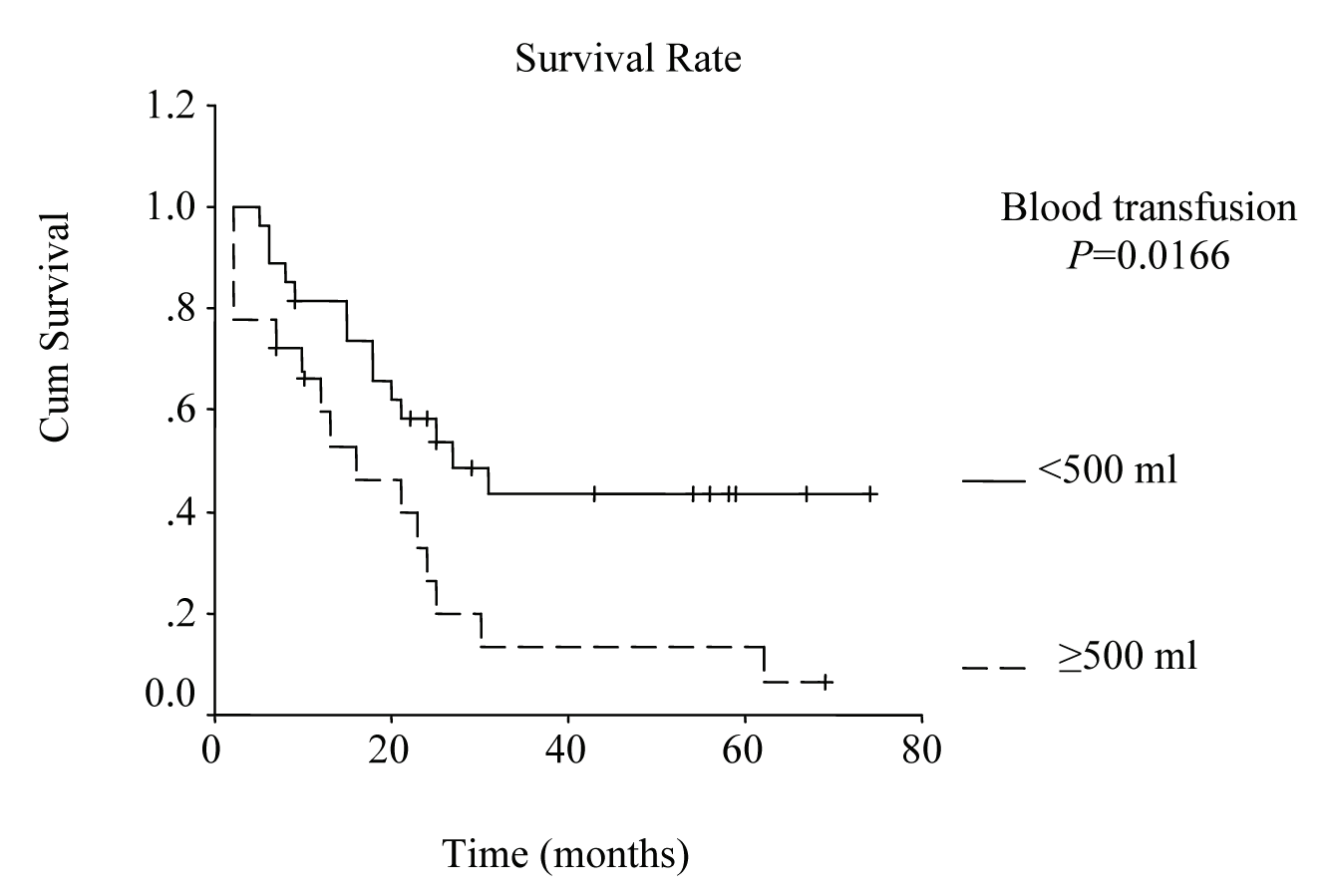
Survival according to blood transfusion. Survival rate for the patients that received blood transfusions more than or equal to 500 ml was lower than the rate for the patients that received less than 500 ml. (P = 0.0166, log-rank test).

**Figure 5:**
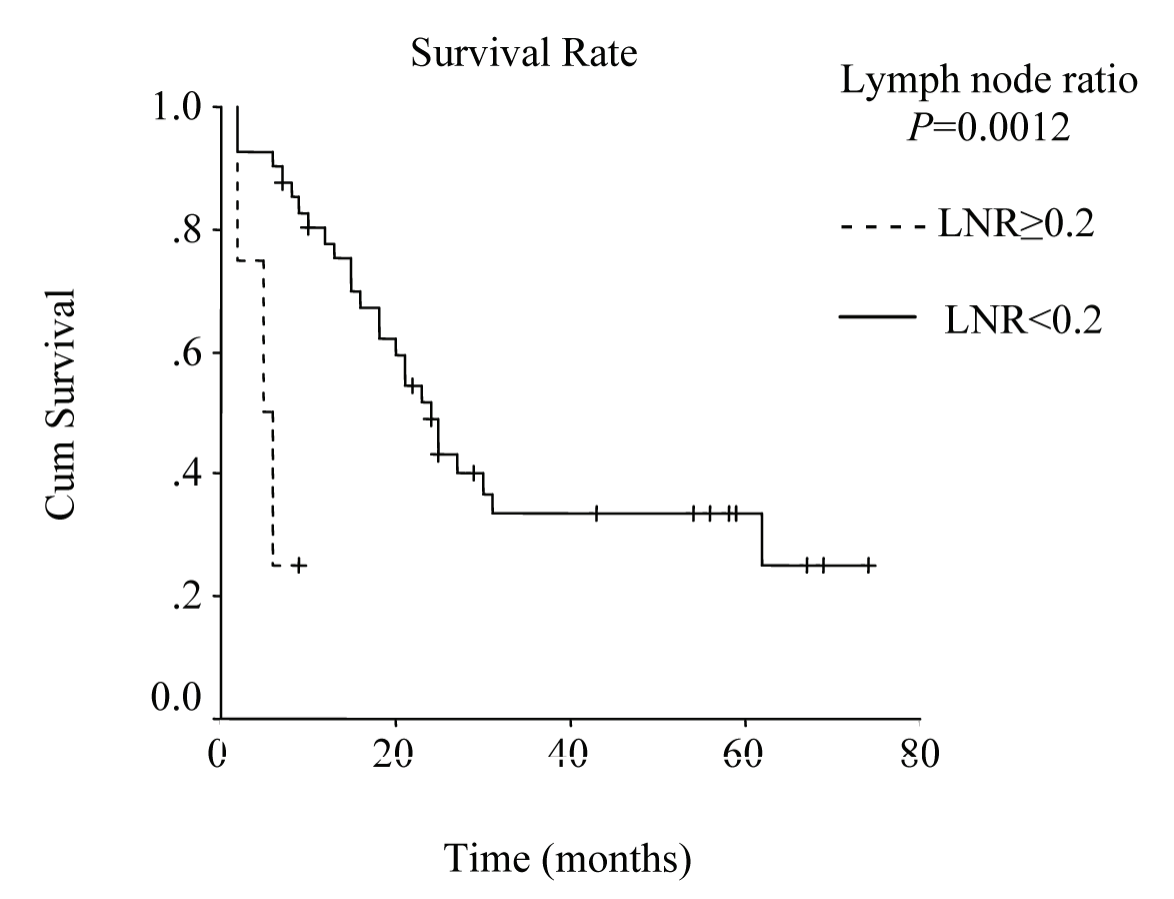
Survival according to lymph node ratio. Survival rate for patients with a lymph node ratio greater than or equal to 0.2 was lower then the rate for those with a lymph node ratio less than 0.2. (P = 0.0012, log-rank test).

The overall survival curve and the survival curves according to lymph node metastases, lymph vessel invasion, blood transfusions, and lymph node ratio are shown in Figure 1 - 5.

## Discussion

Curative surgical treatment of gastric cancer still remains difficult in the Western countries, primarily because most of the patients present with advanced disease [10]. Transmural gastric carcinomas cover a wide range between stages T2N0 and T3N2 with varying survival rates. The extent of the disease, the operative procedure, and patient selection are crucial in optimizing outcome [7]. The standard treatment policy for all potentially curable patients with gastric cancer is radical resection with extensive lymphadenectomy. However, the incidence of complications after gastrectomy increases significantly when extended lymph node dissection is added [7, 11].

Postoperative morbidity and mortality rates for patients who undergo curative gastrectomy are reported to range from 10.5 to 33% and from 2 to 11.9%, respectively [10, 12, 13]. In our series, morbidity and mortality rates were 24% and 10%, respectively. Anastomotic leakage was the most common major postoperative complication (10%). The reported incidence rates for anastomotic leakage after extended gastrectomy varies between 1% and 12.3% [14, 15]. Sasako et al reported that the recent incidence of leakage at the esophago-jejunostomy site after learning curve for stapled anastomosis is less than 1% [11].

There have been reports on minimal positive effects of adjuvant chemotherapy for gastric cancer patients [16]. Results of the large American Southwest Oncology Group (SWOG) study advocate postoperative radio-chemotherapy for T2N0-T3N2 gastric cancer patients. National Intergroup Trial (INT-116), a large study of postoperative radio-chemotherapy with 5-flourouracil/leucoverin and 5-fluorouracil/leucoverin + 45 Gy radiotherapy, showed that adjuvant radio-chemotherapy significantly improves survival [17]. Thus, adjuvant radio-chomotherapy for surgically treated gastric cancer patients is advocated by the oncology group in our hospital as in many other centers [6].

The prognosis for patients with transmural advanced gastric carcinoma has been improved by curative surgery [7]. Several reports have demonstrated that the significant prognostic factors are the stage of the disease, lymph node status, and the depth of penetration into the gastric wall [12, 14, 18]. Five-year survival rate after curative surgical resection ranges from 50% to 70% in patients with stage II disease and from 29% to 42% in patients with stage III disease [10, 12]. In our series, the patients with pT2 and stage IB disease had better survival than those with pT3 and the other stages, but they were not significant variables in univariate analysis. In Dhar’s [7] series 49% of the patients survived more than 7 years whereas 5-year survival rate was 19% in this study. Five-year survival rates indicate the difference between patients in the West and in the East, and that the prognosis of resectable gastric carcinoma in the West remains poor. The survival rate that we obtained in this study is comparable with those from the Western countries but lower than those from Japan. Better results of the Japanese series may be due to early diagnosis, different tumor biology, patient type, and extended lymphadenectomy.

It has been reported that the prognosis of the patients with gastric cancer is influenced most strongly by lymph node involvement. Moreover, lymphatic spread is reported to be one of the most relevant prognostic factors in advanced gastric cancer resected for cure [18, 19]. Since risk of relapse and overall survival are also highly dependent on the number of lymph node metastases, overall survival of the patients with no lymph node metastasis is about 40-80% [20]. In the present study, 5-year survival rate in relation to lymph node involvement was 65%, 13%, and 11% for pN0, pN1, and pN2, respectively and it was one of the significant prognostic factors by univariate analysis (P = 0.030).

Studies that have been published during last few years emphasize the prognostic importance of lymph vessel invasion. Yokota et al found that lymphatic vessel invasion is an unfavorable prognostic factor for gastric cancer [18]. Also, von Rahden et al reported lymphatic vessel invasion as a prognostic factor in patients with resected primary adenocarcinomas of the esophagogastric junction [21]. In our study, lymphatic vessel invasion was found to be one of the prognostic factors by the univariate analysis (P = 0.001).

Multiple blood transfusions have been reported to be associated with poor survival rate, and have been determined as an independent risk factor for poor prognosis by multivariate analysis in several studies [22, 23]. In a retrospective study on 1000 patients that had undergone curative surgery for gastric cancer at the National Cancer Center Hospital in Japan from 1976 to 1981, statistical analysis revealed significant adverse influence of blood transfusions on survival [24]. In our study, blood transfusions more than 500 ml was one of the significant prognostic factors by univariate analysis (P = 0.021).

Lymph node ratio may give more accurate prognostic information about nodal involvement than the current pN category. Several authors have already suggested the role of lymph node ratio as a prognostic factor [14, 25, 26]. Siewert et al reported relevant prognostic factors in 1182 patients with gastric cancer undergoing R0 resection and they noted that lymph node ratio and lymph node status were the most important prognostic factors in patients with resected gastric cancer [26]. Analyzing the survival by comparing lymph node ratio against the number of involved lymph nodes, Santiago et al have demonstrated that the ratio stands out as the best prognostic factor [25]. The results of our study show that lymph node ratio was the only important predictor in patients with transmural gastric cancer (P = 0.029, RR:4.47).

In conclusion, lymph node metastases, lymph vessel invasion, blood transfusions (more than or equal to 500 ml), and lymph node ratio (greater than or equal to 0.2) were found as significant prognostic factors by the univariate analysis in this study. However, lymph node ratio was the strongest predictor of survival in multivariate analysis. Our data showed that we can expect a good survival for patients with a lymph node ratio less than 0.2, when extended lymph node dissection is performed.
